# Epitope-optimization creates highly immunogenic alpha fetoprotein antigen to break immune tolerance and potently activates CD8 T cells to prevents autochthonous hepatocellular carcinoma

**DOI:** 10.1186/2051-1426-1-S1-P216

**Published:** 2013-11-07

**Authors:** Yuan Hong, Yibing Peng, Sheng Z Guo, Junfeng Pang, Jose Guevara-patino, David H Munn, Nahid Mivechi, David Bartlett, Yukai He

**Affiliations:** 1 Cancer Center, Georgia Regents University, Augusta, GA, USA; 2 Surgery, Loyola University, Chicago, IL, USA; 3 Surgery, University of Pittsburgh, Pittsburgh, PA, USA

## 

In this study, we investigated whether mouse alpha fetoprotein (mAFP), the shared self/tumor antigen of hepatocellular carcinoma (HCC), could be rationally engineered to create effective vaccine to break tolerance and potently activate CD8 T cells to prevent clinically-relevant carcinogen-induced autochthonous HCC. We found that the computer-guided epitope-optimization created optimized opt-mAFP and that immunization with lentivector (lv) expressing opt-mAFP, but not wt-mAFP, potently activated CD8 cells specific for three novel H-2b restricted CD8 epitopes, which cross-recognized wt-mAFP epitopes naturally processed and presented by wt-mAFP+ tumor cells. Immunization with opt-mAFP-lv, but not wt-mAFP-lv, completely protected mice from wt-mAFP+ tumor challenge and effectively prevented carcinogen-induced autochthonous HCC. Prime-boost with opt-mAFP-lv and vaccinia vector opt-mAFP-vv significantly increased the wt-mAFP-specific CD8 T cells that were highly responsive to emerging HCC tumor cells in the liver, enhancing prevention of autochthonous HCC. Our data demonstrate that epitope-optimization creates immunogenic opt-mAFP that is able to break tolerance and activate potent CD8 responses, which can cross-recognize wt-mAFP peptides, but also recognize and kill mAFP+ tumor cells. Our study provides a practical roadmap to develop effective human vaccines that should have a better chance of success than the current human HCC vaccines based on native wt-AFP.

